# Incorporation of Mn^2+^ into CdSe quantum dots by chemical bath co-deposition method for photovoltaic enhancement of quantum dot-sensitized solar cells

**DOI:** 10.1098/rsos.171712

**Published:** 2018-03-21

**Authors:** Chenguang Zhang, Shaowen Liu, Xingwei Liu, Fei Deng, Yan Xiong, Fang-Chang Tsai

**Affiliations:** 1 School of Physics and Optoelectronic Engineering, Yangtze University, Jingzhou, Hubei 434023, People's Republic of China; 2 Key Laboratory for the Green Preparation and Application of Functional Materials, Ministry of Education, School of Materials Science and Engineering, Hubei University, Wuhan, Hubei 430062, People's Republic of China

**Keywords:** quantum-dot-sensitized solar cells, chemical bath co-deposition, Mn-doped quantum dots, photoelectric conversion efficiency

## Abstract

A photoelectric conversion efficiency (PCE) of 4.9% was obtained under 100 mW cm^−2^ illumination by quantum-dot-sensitized solar cells (QDSSCs) using a CdS/Mn : CdSe sensitizer. CdS quantum dots (QDs) were deposited on a TiO_2_ mesoporous oxide film by successive ionic layer absorption and reaction. Mn^2+^ doping into CdSe QDs is an innovative and simple method—chemical bath co-deposition, that is, mixing the Mn ion source with CdSe precursor solution for Mn : CdSe QD deposition. Compared with the CdS/CdSe sensitizer without Mn^2+^ incorporation, the PCE was increased from 3.4% to 4.9%. The effects of Mn^2+^ doping on the chemical, physical and photovoltaic properties of the QDSSCs were investigated by energy dispersive spectrometry, absorption spectroscopy, photocurrent density–voltage characteristics and electrochemical impedance spectroscopy. Mn-doped CdSe QDs in QDSSCs can obtain superior light absorption, faster electron transport and slower charge recombination than CdSe QDs.

## 1. Introduction

With the rapid development of the global economy, the demand for energy has continued to increase since the beginning of the twenty-first century. In the last 30 years, solar cells have achieved considerable development and may be regarded as one of the main sources of future power [1]. Dye-sensitized solar cells (DSSCs) have been developed in past decades due to their high absorption, high stability and potential to achieve efficient conversion of sunlight into electricity. The photoelectric conversion efficiency (PCE) of DSSCs based on a planar substrate of a rigid conducting glass has reached greater than 11% [2,3]. Replacing the organic dyes by semiconductor quantum dots (QDs) in sensitizers, quantum-dot-sensitized solar cells (QDSSCs) exhibit the unique advantages of quantum size effect, multi-exciton effect, large absorption coefficient and easy matching of energy levels between the electron donor and acceptor materials [4,5]. QDs, which include CdS, CdSe, CdTe [6], PbS [7], Ag_2_S [8], Ag_2_Se [9], CuInS_2_ [10–12] and CuInSe_2_ [13], are numerous. Lee & Lo used CdS/CdSe co-sensitized TiO_2_ to obtain QDSSC and achieved PCE of up to 4% [14]. Since then, CdS/CdSe has been widely studied as a classical co-sensitization system. The CdS QDs adsorbed on TiO_2_ films show a good effect on the deposition of CdSe QDs, finally, forming a classical TiO_2_/CdS/CdSe cascade structure. Santra & Kamat doped Mn^2+^ into CdS QDs, thus obtaining a considerable increase in PCE of Mn : CdS/CdSe-sensitized solar cells [15]. Although QDSSC PCE still currently lags behind the maximum efficiency of 15% obtained by DSSCs, the gap has been rapidly reduced, thereby resulting in QDSSC PCE of approximately 9% [16–21].

Adopting the DSSC principle, QDSSCs are generally comprised of a QD sensitizer, mesoporous oxide including titanium dioxide (TiO_2_) or zinc oxide (ZnO), polysulfide electrolyte as a redox couple, and Cu_2_S as a counter electrode. Despite many efforts devoted to QDSSCs, the cell efficiency still remains less than 10% [21,22]. An important reason for this moderate efficiency is the inferior optoelectronic properties of QD sensitizers. Moreover, TiO_2_ nanocrystals are stacked in the film, and the photoelectrons are subjected to a large number of grain boundary potentials during transmission, which slows down the transmission rate of the photoelectrons in the film. Meanwhile, photoelectrons are easily captured during transmission due to the existence of abundant surface defects on the nanoparticle surface, which increases the probability of photoelectron recombination. Therefore, introducing transition metal ion dopants, such as Mn^2+^, is a promising strategy to modify the intrinsic QD properties and reduce the possibility of photoelectron recombination [23]. CdSe QDs are more attractive due to their high light-harvesting capability in the visible region than CdS and PbS QDs [21,24,25]. The introduction of metal ions into CdSe QDs is useful for achieving superior photovoltaic (PV) performance in QDSSCs. Doping transition metal ions into CdS/CdSe QDs would lead to new materials showing extraordinary electronic and photo-physical properties of QDs. Mn has been doped in QDs to improve the performance of as-prepared materials, such as phosphorescent nanosensor and signal-generation tags for photoelectrochemical immunoassay [26,27]. Mn-doped QDs were also used in QDSSCs, and researchers commonly used successive ionic layer absorption and reaction (SILAR) method to synthesize Mn ions to the CdSe QD surface [23,28]. Wang *et al*. reported Mn : CdSeTe QDs which were prepared by dissolving Mn with oleic acid and paraffin mixed with high-temperature nitrogen as the reaction system [29]. In this study, Mn : CdSe QDs were prepared by chemical bath co-deposition method, that is, mixing the Mn ion source with CdSe precursor solution for QD deposition. Doped Mn^2+^ alters the inherent QD properties, thereby changing the charge separation and combination and increasing the light-harvesting capability. In addition, the chemical bath co-deposition method is easy to operate and accurately controls Mn attachment to the CdSe QDs. The results show that Mn^2+^-doped CdSe QDs exhibit a positive effect on light harvesting and the capability of charge transfer and collection, thus further enhancing the PV performance of QDSSCs. Consequently, the CdS/Mn : CdSe QDSSCs exhibited high PCE of 4.9% under simulated illumination of 100 mW cm^−2^.

## 2. Experimental set-up

### 2.1. Materials

CdS QDs were prepared using sodium sulfide nonahydrate (Na_2_S·9H_2_O ≥ 98%) and cadmium nitrate tetrahydrate (Cd(NO_3_)_2_·4H_2_O ≥ 98.0%).

Mn^2+^-doped CdSe QDs were prepared using manganese(II) acetate tetrahydrate (Mn(CH_3_COO)_2_·4H_2_O ≥ 99.0%), selenium (Se ≥ 99.5%), sodium sulfite (Na_2_SO_3_ ≥ 98.0%), cadmium sulfate hydrate (CdSO_4_·8/3H_2_O ≥ 99.0%), nitrilotriacetic acid (C_6_H_9_NO_6_ ≥ 99.0%) and potassium hydroxide (KOH ≥ 85.0%). All chemicals were commercially available and of analytical grade.

### 2.2. Preparation of CdS/CdSe and CdS/Mn : CdSe photoanode

TiO_2_ films with a particle diameter of 20 nm were prepared by screen printing to an effective area of 0.16 cm^2^ on a pre-cleaned fluorine-doped tin oxide (FTO) glass, followed by annealing at 450°C for 30 min in a muffle furnace. For CdS QDs, a TiO_2_ film was dipped into an ethanol solution containing 0.1 M Cd(NO_3_)_2_ for 1 min, rinsed with ethanol, and then dipped for another 1 min into a 0.1 M Na_2_S methanol solution and rinsed again with methanol. The two-step dipping procedure is regarded as one SILAR cycle, and the incorporated amount of CdS can be increased by repeating the assembly cycles. A total of 12 cycles were performed, followed by drying in air [30].

Subsequently, by using nitriloacetate as a complex and selenosulfate as Se source, CdSe was deposited by chemical bath deposition. First, for the Se source, Na_2_SeSO_3_ aqueous solution was freshly prepared by refluxing 0.2 M Se powder in an aqueous solution of 0.5 M Na_2_SO_3_ at 70°C for approximately 5 h. Then, 80 mM CdSO_4_, 160 mM Na_3_NTA and 80 mM Na_2_SeSO_3_ were mixed. TiO_2_ electrodes adsorbed with CdS QDs were placed in a glass container filled with the final solution at room temperature in the dark for 4 h to promote CdSe QD adsorption.

A molar percentage of 10% Mn(CH_3_COO)_2_ was mixed with CdSO_4_ before CdSe deposition to incorporate Mn^2+^. The TiO_2_/CdS electrode was immersed in the mixed solution and placed in the dark at room temperature for 4 h. After removal, the anode was washed with deionized water and dried in air.

### 2.3. Quantum-dot-sensitized solar cell assembly and characterization

The sensitized TiO_2_ films were used as photoanodes with compact Cu_2_S film as the counter electrode. The electrolyte, which comprised 1 M Na_2_S, 0.1 M S and 0.2 M KCl in a water/methanol (1 : 1 by volume) solution, was injected between the photoanode and counter electrode through siphonic effect.

The PV performances, which include short-circuit current density (*J*_sc_), open-circuit voltage (*V*_oc_), fill factor (FF) and power conversion efficiency (*η*), of the cells were examined by measuring the current density–voltage (*J*–*V*) characteristics of the cells using a Keithley 2450 source meter under a light intensity of 100 mW cm^−2^ offered by a xenon lamp (300 W; Nbet, HSX-F300). The optical absorption spectra were measured by a spectrophotometer (Shimadzu, UV-2450). The QD microstructure was analysed with a field emission scanning electronic microscope (SEM; JEOL, JSM7100F) and a transmission electron microscope (TEM; JEM 2100F STEM/EDS). Electrochemical impedance spectroscopy (EIS) measurements were obtained using an electrochemical workstation (CorrTest, CS 350H). Elemental analysis was conducted with an energy dispersive spectrometer (EDS; Oxford X-MAX).

## 3. Results and discussion

**Figure 1.**
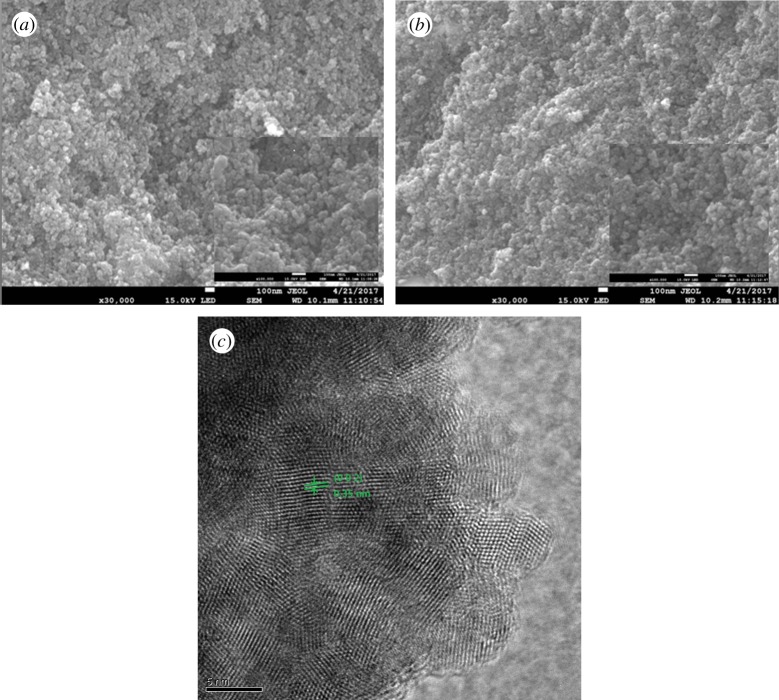
SEM images of (*a*) CdS/CdSe and (*b*) CdS/Mn : CdSe QD sensitization on TiO_2_ surface. (*c*) TEM image of CdS/Mn : CdSe QDs.

Figure 1*a*,*b* shows the SEM images of the CdS/CdSe and CdS/Mn : CdSe QDs deposited on the TiO_2_ surface. The structure of the TiO_2_ film, which is composed of TiO_2_ nanoparticles of approximately 20–30 nm, is loose and porous. This porous structure facilitates the permeation of precursor fluid into the film for depositing QDs. Figure 1*a* shows the evenly distributed nanoparticles on the film with a diameter in the range of approximately 25–45 nm. When Mn^2+^ is doped into the CdSe as shown in figure 1*b*, the QDs on the surface of the film are compact and the voids among the particles are small, thus reducing the recombination of photogenerated electrons, which is beneficial to the improvement of the overall photoelectric efficiency of the solar cells. With Mn^2+^ loading into the CdSe, the size of the QD clusters is increased. Figure 1*c* shows the TEM image of CdS/Mn : CdSe QDs to present the morphology of QD.

**Figure 2.**
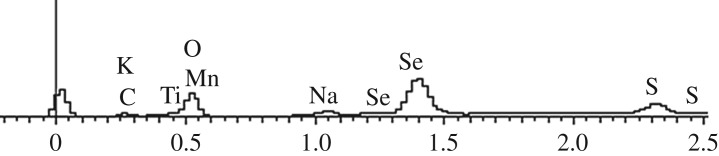
Energy spectrum of FTO/TiO_2_/CdS/Mn : CdSe photoelectrode.

EDS analysis was conducted to investigate the elemental compositions of CdS/Mn : CdSe QD sensitizers on top of TiO_2_. The results are shown in figure 2 and table 1. The samples were characterized by O, Ti, Cd, Se and a small amount of S and Mn, which indicates the existence of TiO_2_, CdSe and CdS in the sample. In addition, the presence of Mn indicates that Mn^2+^ is indeed incorporated into CdSe QDs. A small amount of other elements (e.g. C, Na and K) may occur during mixing of impurities or pharmaceutical impurities.

**Table 1.** Element distribution of FTO/TiO_2_/CdS/Mn : CdSe photoelectrode in EDS analysis.

	C–K	O–K	Na–K	S–K	Ti–K	K–K	Mn–K	Se–L	Cd–L	
weight percentage	3.01	30.22	1.07	1.59	29.93	0.54	1.45	10.85	21.34	100
atomic percentage	7.74	58.39	1.44	1.53	19.31	0.42	1.05	4.25	5.87	100

**Figure 3.**
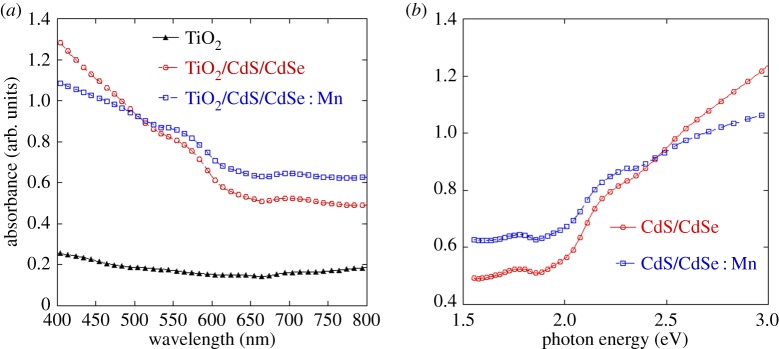
UV–visible absorption spectra of bare TiO_2_, TiO_2_/CdS/CdSe and TiO_2_/CdS/Mn : CdSe.

In solar cells, the UV–visible absorption spectrum was used to measure the absorptive capacity of the absorptive layer material. Figure 3*a* displays the UV–visible spectral curves of TiO_2_ films loaded with CdS/CdSe and CdS/Mn : CdSe QDs. The result shows that the absorbance of the CdS/Mn : CdSe QDs is higher than that of the CdS/CdSe QDs. The light absorption intensity of CdS/CdSe QDs is higher than that of CdS/Mn : CdSe QDs in the wavelength region of less than 500 nm, and this finding is in agreement with [23]. However, when the wavelength is between 500 nm and 800 nm, the light absorption intensity of CdS/CdSe QDs is weaker than that of CdS/Mn : CdSe QDs. In general, CdS/Mn : CdSe QDs show a relatively stable absorption within 400–800 nm, thereby indicating that the absorption capacity of CdS/Mn : CdSe QDs is stronger than that of CdS/CdSe QDs. This result corresponds to the increase in current density of the Mn-doped solar cell. The high absorbance of the photoelectrode might be attributed to the effects of Mn^2+^ doped into CdS/CdSe QDs and a high loading amount of QDs. Furthermore, the band gap of semiconductor could be inferred from the absorption edge. It can be estimated from figure 3*b* that the band gap of CdSe QDs is 1.87 eV, and the band gap of Mn-doped QDs is 1.73 eV. Thus, the incorporation of Mn^2+^ ion into CdSe narrows the band gap of QDs.

**Figure 4.**
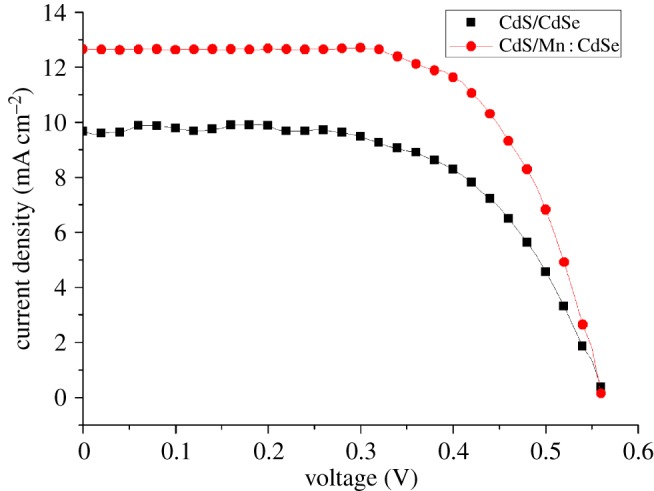
*J*–*V* curves of QDSSCs based on CdS/CdSe and CdS/Mn : CdSe QDs.

Figure 4 shows the *J*–*V* characteristics of the solar cells. Table 2 shows the key parameters (*J*_sc_, *V*_oc_, FF and maximum *η*) of CdS/CdSe and CdS/Mn : CdSe QDSSCs. For devices based on two different photoanodes, there is slight difference between the *V*_oc_. However, the CdS/Mn : CdSe device shows a higher *J*_sc_ (12.65 mA cm^−2^) than CdS/CdSe device (9.67 mA cm^−2^), thereby resulting in a higher cell efficiency (4.9%) and FF (0.58) than the solar cell based on CdS/CdSe QDs (*η*_max_ of 3.4% and FF of 0.51). Doping Mn^2+^ in QDSSCs significantly improves the photoanode. As previously mentioned, when Mn^2+^ is incorporated into CdSe, the absorption curve is observed to elevate within the visible wavelengths of 500–800 nm, and a cascade energy level is formed which is favourable for charge transport inside the solar cell [23], which reduces the recombination of electrons and holes and improves the light-harvesting capability of the photoanode. Thus, the photocurrent and the PCE are improved.

**Table 2.** Photovoltaic parameters of QDSSCs based on CdS/CdSe and CdS/Mn : CdSe QDs.

QDs	*V*_oc_ (V)	*J*_sc_ (mA cm^−2^)	FF	*η*_max_ (%)
CdS/CdSe	0.56	9.67	0.51	3.4
CdS/Mn : CdSe	0.57	12.65	0.58	4.9

**Figure 5.**
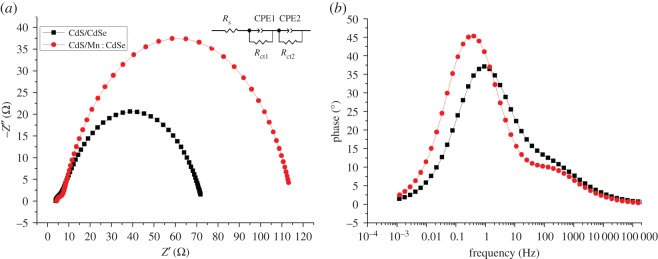
(*a*) Nyquist plot and (*b*) Bode plot of the QDSSCs under 100 mW cm^−2^ illumination and the frequency ranging from 0.1 Hz to 500 kHz at room temperature. Inset: equivalent circuit model of the QDSSCs.

In the QDSSC study, EIS is a useful tool for obtaining the series resistance (*R*_s_), the load resistance (*R*_ct_), the ion diffusion resistance (*Z*_w_), the interface double layer chemical capacitance (CPE) and other parameter information [21,31,32]. Figure 5*a* shows the Nyquist plots of QDSSCs under illumination of 100 mW cm^−2^, and there are two semicircles which could be fitted with an equivalent circuit as shown in the inset of figure 5*a* [33,34].

The equivalent circuit is composed of series resistance *R*_s_, transfer resistance *R*_ct1_ and *R*_ct2_ and chemical capacitance CPE_1_ and CPE_2_. *R*_s_ is ascribed to the contact resistance of FTO/TiO_2_. *R*_ct1_ is ascribed to the charge transfer resistance at the interface of the electrolyte/counter electrode and *R*_ct2_ is ascribed to the charge transfer resistance at the interface of TiO_2_/QD/electrolyte. CPE_1_ and CPE_2_ are constant phase elements of the capacitance corresponding to *R*_ct1_ and *R*_ct2_, respectively. The data of the equivalent circuit are listed in table 3. Compared with the classical CdS/CdSe structure, the CdS/Mn : CdSe structure shows a smaller charge resistance, which indicates that the electrons at the TiO_2_/QD/electrolyte interface rapidly transfer. An increased charge transfer resistance leads to a decreased electron transfer rate and poor efficiency. The low *R*_ct_ value is favourable for electron transport, which ensures a minimal diffusion obstruction when the electrons travel over a long distance. This phenomenon may lead to the reduction of electronic recombination and lifetime growth.

**Table 3.** Parameters obtained by fitting the impedance spectra of QDSSCs using the equivalent circuit in figure 5.

cell	*R*_s_ (Ω)	*R*_ct1_ (Ω)	*R*_ct2_ (Ω)	CPE_1_ (μF)	CPE_2_ (μF)
CdS/CdSe	8.677	2.31	140.10	0.603	0.682
CdS/Mn : CdSe	2.663	1.91	96.51	0.924	0.812

The Bode diagrams are shown in figure 5*b*. The lifetime *τ* of the injected electrons in the TiO_2_ photoanode is related to the position of the mid-frequency peak *f*_max_, which is defined as follows:

3.1 $$\tau =\frac{1}{2\pi f_{\max}},$$

where *f*_max_ means the frequency of superimposed alternating current voltage [35]. The *τ* value of CdS/Mn : CdSe device is higher (0.48 ms) than that of CdS/CdSe (0.17 ms), which indicates that Mn^2+^ doping into CdSe QDs leads to a longer electron lifetime of CdS/CdSe QDSSCs. This finding can be attributed to the doping of Mn^2+^, which may change and optimize QDs of the surface or interface structure, thereby reducing the *R*_ct_ value and the recombination of electrons during transmission [23]. This result is in agreement with the PV characteristic.

## 4. Conclusion

By mixing the Mn ion source with CdSe precursor solution, the Mn : CdSe QDs were deposited and prepared into QDSSCs. Mn^2+^ introduction into the CdSe QDs by this method improves the light harvest and charge transfer. In the CdS/Mn : CdSe QDs, the increase of the electron-collecting efficiency leads to a PCE improvement of the QDSSCs of up to 4.9%. Based on EIS analysis, the electron lifetime in CdS/Mn : CdSe devices is higher than that in devices based on CdS/CdSe, which indicates that the probability of charge recombination at the interface decreases due to the presence of Mn^2+^. Incorporation of Mn^2+^ into CdSe QD by the proposed chemical bath co-deposition method shows excellent photoelectric properties. This method is also proposed for effective QDSSC preparation.

## Data accessibility

Our data are deposited at Dryad (http://dx.doi.org/10.5061/dryad.27g26) [36].
